# HSP70 Inhibitor Suppresses IGF-I-Stimulated Migration of Osteoblasts through p44/p42 MAP Kinase

**DOI:** 10.3390/biomedicines6040109

**Published:** 2018-11-21

**Authors:** Tetsu Kawabata, Haruhiko Tokuda, Go Sakai, Kazuhiko Fujita, Rie Matsushima-Nishiwaki, Gen Kuroyanagi, Takanobu Otsuka, Osamu Kozawa

**Affiliations:** 1 Department of Orthopedic Surgery, Nagoya City University Graduate School of Medical Sciences, Nagoya 467-8601, Japan; tetsu0512.misa0731@kg7.so-net.ne.jp (T.K.); sakaigo_sakaigo@yahoo.co.jp (G.S.); picorukazoo@yahoo.co.jp (K.F.); kokuryugen@yahoo.co.jp (G.K.); t.otsuka@med.nagoya-cu.ac.jp (T.O.); 2 Department of Pharmacology, Gifu University Graduate School of Medicine, Gifu 501-1194, Japan; tokuda@ncgg.go.jp (H.T.); riemn@gifu-u.ac.jp (R.M.-N.); 3 Department of Orthopedic Surgery, Toyokawa City Hospital, Toyokawa 442-8561, Japan; 4 Department of Clinical Laboratory, National Center for Geriatrics and Gerontology, Obu 474-8511, Japan; 5 Department of Rehabilitation Medicine, Nagoya City University Graduate School of Medical Sciences, Nagoya 467-8601, Japan

**Keywords:** migration, HSP70 inhibitor, IGF-I, p44/p42 MAP kinase, osteoblast

## Abstract

Heat shock protein 70 (HSP70) is a ubiquitously expressed molecular chaperone in a variety of cells including osteoblasts. We previously showed that insulin-like growth factor-I (IGF-I) elicits migration of osteoblast-like MC3T3-E1 cells through the activation of phosphatidylinositol 3-kinase/Akt and p44/p42 mitogen-activated protein (MAP) kinase. In the present study, we investigated the effects of HSP70 inhibitors on the IGF-I-elicited migration of these cells and the mechanism involved. The IGF-I-stimulated osteoblast migration evaluated by a wound-healing assay and by a transwell cell migration was significantly reduced by VER-155008 and YM-08, which are both HSP70 inhibitors. VER-155008 markedly suppressed the IGF-I-induced phosphorylation of p44/p42 MAP kinase without affecting that of Akt. In conclusion, our results strongly suggest that the HSP70 inhibitor reduces the IGF-I-elicited migration of osteoblasts via the p44/p42 MAP kinase.

## 1. Introduction

It is firmly established that bone metabolism is regulated cooperatively by bone-forming osteoblasts and bone-resorbing osteoclasts and that bone tissue is consistently regenerated through bone remodeling [1,2]. The process of bone remodeling is initiated with osteoclastic bone resorption and osteoblasts subsequently migrate to the resorbed sites, which leads to bone formation. Adequate bone mass is maintained by the orchestrated cooperation of osteoclasts and osteoblasts [2]. Thus, the impairment of bone remodeling causes metabolic bone diseases such as osteoporosis. Evidence is accumulating that osteoblast migration is essential not only for physiological bone metabolism but also for pathological bone processes including bone-fracture healing [1,3,4,5]. However, the exact mechanism behind osteoblast migration has not yet been clarified.

Insulin-like growth factor-I (IGF-I), which is embedded abundantly in the bone matrix, plays a crucial role in the regulation of bone metabolism [6,7]. Regarding IGF-I-effects on osteoblasts, we have previously shown that IGF-I upregulates the activity of alkaline phosphatase, which is a biomarker of bone formation, via p44/p42 mitogen-activated protein (MAP) kinase and phosphatidylinositol 3-kinase/Akt in osteoblast-like MC3T3-E1 cells [8,9]. As for the effect of IGF-I on osteoblast migration, IGF-I activates Akt and stimulates migration of osteoblast-like MC3T3-E1 cells as a chemotactic factor [10]. In our study [11], we have demonstrated that p44/p42 MAP kinase and phosphatidylinositol 3-kinase/Akt act as positive regulators in the IGF-I-induced migration of osteoblast-like MC3T3-E1 cells. However, how the molecular mechanism underlying IGF-I induces the osteoblast migration is unknown.

Heat shock proteins (HSPs) are induced in the cells exposed to various environmental stresses such as heat, hypoxia, and oxidation [12]. It is firmly established that HSPs play an important role as molecular chaperones in proteostasis under stress conditions [12]. Among HSPs, it is known that HSP70 (HSPA) is constitutively expressed in the unstressed cells and that HSP70 is involved in various physiological cell functions such as the regulation of steroid hormone receptors [13]. On the other hand, accumulating evidence indicates that HSP70 plays a pivotal role in pathological conditions including cancer, infection, and autoimmune diseases [14]. It has been reported that the overexpression of the HSP70 protein in tumor tissue is related to worse outcomes [15]. Therefore, it is currently recognized that the suppression of the HSP70 function is one possible therapeutic target against these diseases. With regard to the effects of HSP70 on bone cells, extracellular HSP70 reportedly stimulates the alkaline phosphatase activity and induces mineralization of human mesenchymal stem cells [16]. However, the details of HSP70 in osteoblasts remain to be clarified.

In the present study, we investigated the effects of HSP70 inhibitors on the IGF-I-elicited migration of osteoblast-like MC3T3-E1 cells and the underlying mechanism. In this paper, we show that the HSP70 inhibitor suppresses the IGF-I-elicited migration of osteoblasts through attenuation of the p44/p42 MAP kinase pathway.

## 2. Materials and Methods

### 2.1. Materials

IGF-I was obtained from R&D System, Inc. (Minneapolis, MN, USA). VER-155008 and YM-08 were obtained from Sigma-Aldrich Co. (St. Louis, MO, USA). Phospho-specific p44/p42 MAP kinase, p44/p42 MAP kinase, phospho-specific Akt (Thr308), and Akt antibodies were used for the first antibodies (Cell Signaling, Beverly, MA, USA). An ECL Western blotting detection kit was used (GE Healthcare UK Ltd., Buckinghamshire, UK). Other materials were purchased from commercial sources. VER-155008 and YM-08 were dissolved in dimethyl sulfoxide (DMSO). The maximum concentration of DMSO was 0.1%, which did not affect the assay for cell migration and the detection of the protein level using Western blotting.

### 2.2. Cell Culture

Cloned osteoblast-like MC3T3-E1 cells from an immortalized clonal cell line established from neonatal mouse calvaria [17], which were generously provided by Dr. M. Kumegawa, were maintained as previously reported [18]. MC3T3-E1 cells were cultured in α-minimum essential medium (α-MEM) with 10% fetal bovine serum (FBS) at 37 °C in a humidified atmosphere of 5% CO_2_/95% air. The cells were seeded into 90-mm diameter dishes (2 × 10^5^ cells/dish) in α-MEM containing 10% FBS for five days. The medium was then exchanged for α-MEM containing 0.3% FBS and the cells were subsequently used for Western blot analysis after 48 h. For the cell migration assay, MC3T3-E1 cells were cultured in α-MEM with 10% FBS for three days, sub-cultured in α-MEM with 0.3% FBS for 6 h, and then used for the migration experiments.

### 2.3. Cell Migration Assay

For a wound-healing assay, cultured MC3T3-E1 cells were seeded at 10 × 10^4^ cells/well into an Ibidi Culture-Insert 2 Well (Ibidi, Martinsried, Germany) with a 500-µm margin from the side of the well and allowed to grow for 24 h. After the insert was removed, the cells were then stimulated by 70 nM of IGF-I for 8 h. The cells were visualized by the EOS Kiss X4 digital camera (Canon, Tokyo, Japan) connected to a CK40 culture microscope (Olympus Optical Co. Ltd., Tokyo, Japan) before the stimulation of IGF-I and after 8 h. The area of migrated cells was measured by ImageJ software (version 1.48, NIH, Bethesda, MD, USA).

A transwell cell migration assay was performed by using a Boyden chamber (polycarbonate membrane with 8-µm pores, Transwell^®^ Corning Costar Corp, Cambridge, MA, USA), which was previously described [19]. Cultured MC3T3-E1 cells were trypsinized and seeded (10 × 10^4^ cells/well) onto the upper chamber in α-MEM containing 0.3% FBS. IGF-I (10 nM) was added to the lower chamber in α-MEM with 0.3% FBS and incubated for 16 h at 37 °C. We, then, mechanically removed the cells on the upper surface of the membrane. Cells adherent to the underside of the transwell membrane were fixed with 4% paraformaldehyde and stained with 4′,6-diamidino-2-phenylindole solution. These cells were stained, visualized, and counted by using fluorescent microscopy at a magnification of 20× by counting the stained cells. The migrated cells were photographed and counted by using fluorescent microscopy at a magnification of 20× by counting the stained cells from three randomly chosen high-power fields. When indicated, the cells were pretreated with VER-155008 or YM-08 for 60 min.

### 2.4. Western Blot Analysis

Cultured osteoblast-like MC3T3-E1 cells were pretreated with various doses of VER-155008 for 60 min and then stimulated by 10 nM of IGF-I or vehicle in 1 mL of α-MEM with 0.3% FBS for the indicated periods. The cells were then lysed, homogenized, and sonicated in a lysis buffer containing 62.5 mM Tris/HCl, pH 6.8, 2% sodium dodecyl sulfate (SDS), 50 mM dithiothreitol, and 10% glycerol. SDS-polyacrylamide gel electrophoresis (PAGE) was performed by using the method of Laemmli [20] in 10% polyacrylamide gels. The protein was fractionated and transferred onto an Immun-Blot polyvinyl difluoride (PVDF) membrane (Bio-Rad, Hercules, CA, USA). The membranes were blocked with 5% fat-free dry milk in Tris-buffered saline-Tween (TBS-T, 20 mM Tris/HCl, pH 7.6, 137 mM NaCl, 0.1% Tween 20) for 1 h before incubation with the indicated primary antibodies. Western blot analysis was performed, as described previously [21], using phospho-specific p44/p42 MAP kinase, p44/p42 MAP kinase, phospho-specific Akt or Akt antibodies as primary antibodies with peroxidase-labeled antibodies raised in goat against rabbit IgG (KPL, Inc., Gaithersburg, MD, USA), which are being used as secondary antibodies. The primary and secondary antibodies were diluted to optimal concentrations with 5% fat-free dry milk in TBS-T. The peroxidase activity on the PVDF membrane was visualized on X-ray films by utilizing an ECL Western blotting detection system.

### 2.5. Densitometric Analysis of Western Blotting

A densitometric analysis of Western blotting was performed by using a scanner and Image J software (image J version 1.48, NIH, Bethesda, MD, USA). The background-subtracted signal intensity of each phosphorylation signal was normalized to the respective total protein signal and plotted as the fold increase in comparison to control cells without stimulation.

### 2.6. Statistical Analysis

One-way ANOVA followed by Bonferroni’s post-hoc comparisons tests were performed in all statistical analysis and *p* < 0.05 was considered to be statistically significant. Analysis was carried out by triplicate determinations from three independent cell cultures. All data are presented as the mean ± standard error of the mean (SEM).

## 3. Results

### 3.1. Effect of VER-155008 on the IGF-I-Stimulated Migration of MC3T3-E1 Cells

**Figure 1:**
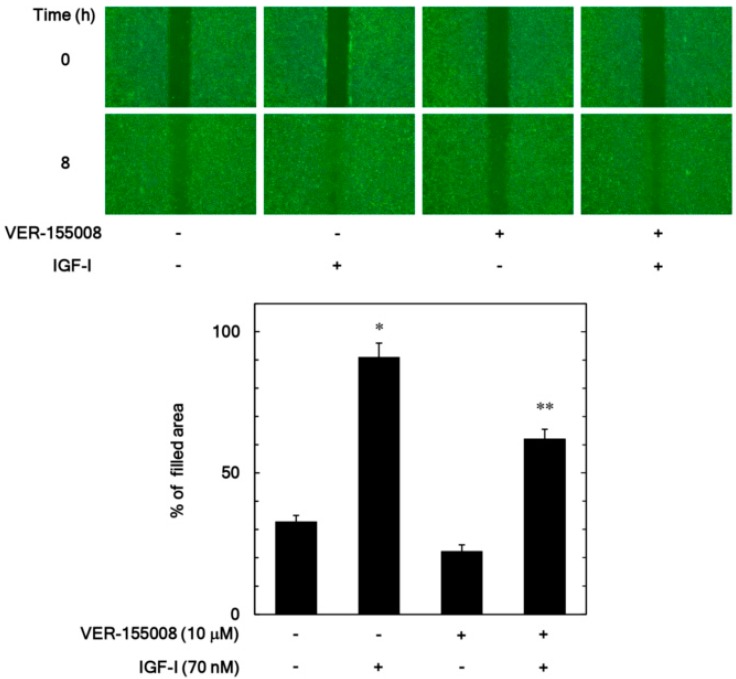
Effect of VER-155008 on the insulin-like growth factor-I (IGF-I)-induced migration of MC3T3-E1 cells. The migration was evaluated by a wound-healing assay. The cells were pretreated with 10 µM of VER-155008 or vehicle for 60 min and then stimulated by 70 nM of IGF-I or vehicle for 8 h. The cells were photographed before IGF-I-stimulation (0 h) and, after 8 h (upper panel, a representative result), the area of migrated cells was measured (lower bar graph), which were obtained from triplicate independent experiments. * *p* < 0.05 compared to the value of the control cells without IGF-I stimulation. ** *p* < 0.05 compared to the value of IGF-I alone.

In our previous study [11], we have shown that IGF-I elicits migration of osteoblast-like MC3T3-E1 cells evaluated by a wound healing assay and a transwell assay. We first examined the effect of VER-155008, an inhibitor of HSP70 [22], on the IGF-I-stimulated migration of MC3T3-E1 cells by a wound-healing assay. The increase of the filled area induced by IGF-I was significantly suppressed by VER-155008 (10 µM), which caused approximately a 35% decrease in the IGF-I-effect (Figure 1).

**Figure 2:**
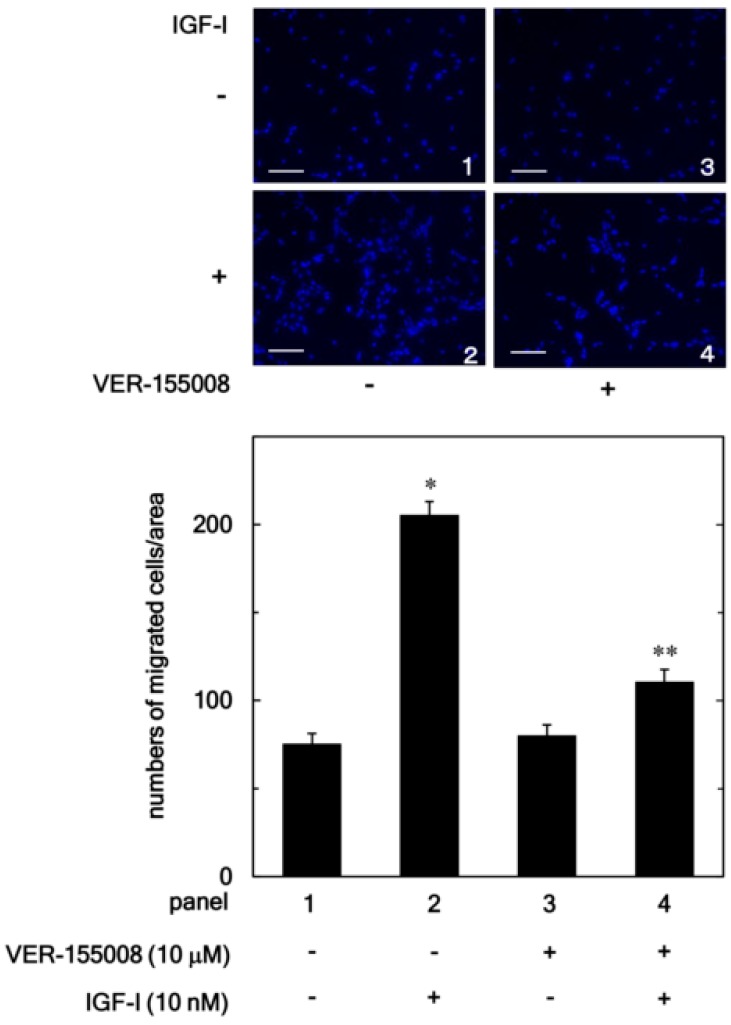
Effect of VER-155008 on the IGF-I-induced migration of MC3T3-E1 cells. The migration was evaluated by using a Boyden chamber. The cells were pretreated with 10 µM of VER-155008 for 60 min and then stimulated by 10 nM of IGF-I or vehicle for 16 h. The representative photographs from triplicate independent experiments and the histogram showing the numbers of migrated cells in each panel are presented. The blue spots indicate nucleus of migrated osteoblast stained by DAPI. * *p* < 0.05 compared to the value of the control cells without IGF-I stimulation. ** *p* < 0.05 compared to the value of IGF-I alone. Scale bar: 100 µm.

In addition, we examined the effect of VER-155008 on the IGF-I-stimulated migration of osteoblast-like MC3T3-E1 cell using a Boyden chamber. VER-155008 markedly reduced the IGF-I-stimulated MC3T3-E1 cell migration (Figure 2). VER-155008 (10 µM) led to an about 75% reduction in the IGF-I-effect.

### 3.2. Effect of YM-08 on the IGF-I-Stimulated Migration of MC3T3-E1 Cells

**Figure 3:**
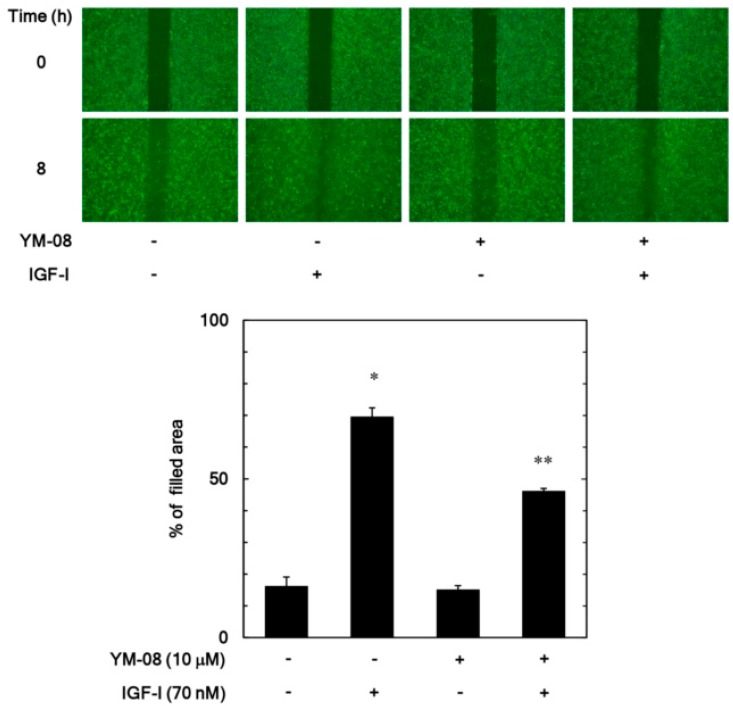
Effect of YM-08 on the IGF-I-induced migration of MC3T3-E1 cells. The migration was evaluated by a wound-healing assay. The cells were pretreated with 10 µM of YM-08 or vehicle for 60 min and then stimulated by 70 nM of IGF-I or vehicle for 8 h. The cells were photographed before IGF-I-stimulation (0 h) and after 8 h (upper panel, a representative result) and the area of migrated cells were measured (lower bar graph), which were obtained from triplicate independent experiments. * *p* < 0.05 compared to the value of the control cells without IGF-I stimulation. ** *p* < 0.05 compared to the value of IGF-I alone.

We next examined the effect of YM-08, which is another inhibitor of HSP70 [23], on the IGF-I-stimulated migration of MC3T3-E1 cells by a wound-healing assay. YM-08 (10 µM) reduced remarkably the increase of the filled area induced by IGF-I (Figure 3). YM-08 (10 µM) led to about 40% reduction in the IGF-I-effect.

**Figure 4:**
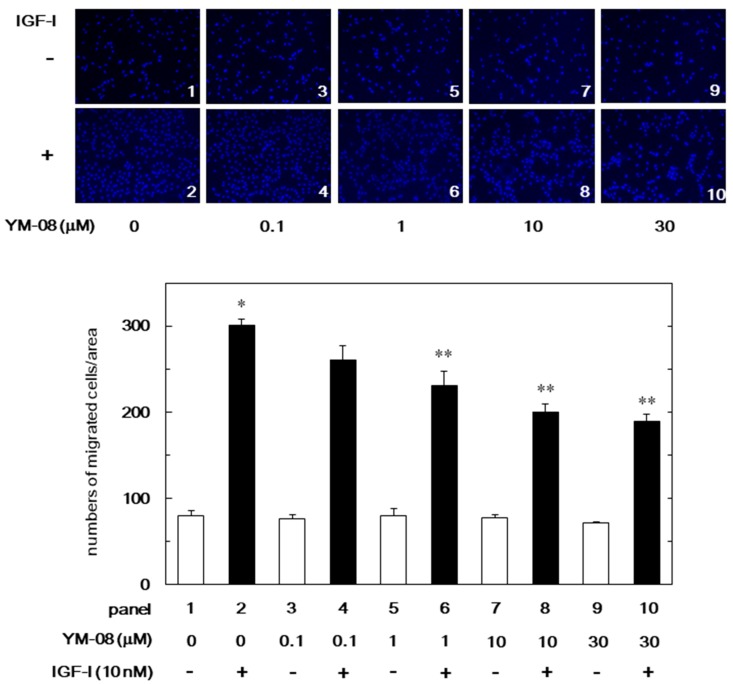
Effect of YM-08 on the IGF-I-induced migration of MC3T3-E1 cells. The migration was evaluated by using a Boyden chamber. The cells were pre-treated with various doses of YM-08 for 60 min and then stimulated by 10 nM of IGF-I or vehicle for 16 h. The representative photographs from triplicate independent experiments and the histogram showing the numbers of migrated cells in each panel are presented. The white and black bars indicate IGF (−) and IGF (+), respectively. The blue spots indicate nucleus of migrated osteoblast stained by DAPI. * *p* < 0.05 compared to the value of the control cells without IGF-I stimulation. ** *p* < 0.05 compared to the value of IGF-I alone. Scale bar: 100 µm.

Additionally, we examined the effect of YM-08 on the IGF-I-stimulated migration of MC3T3-E1 cell using a Boyden chamber. The IGF-I-stimulated migration of cells was significantly decreased by YM-08 (Figure 4). The inhibitory effect of YM-08 on the migration was dose-dependent in the range between 0.1 and 30 µM. YM-08 (30 µM) caused approximately 50% reduction in the IGF-I-effect.

### 3.3. Effects of VER-155008 on the IGF-I-Induced Phosphorylation of p44/p42 MAP Kinase or Akt in MC3T3-E1 Cells

**Figure 5:**
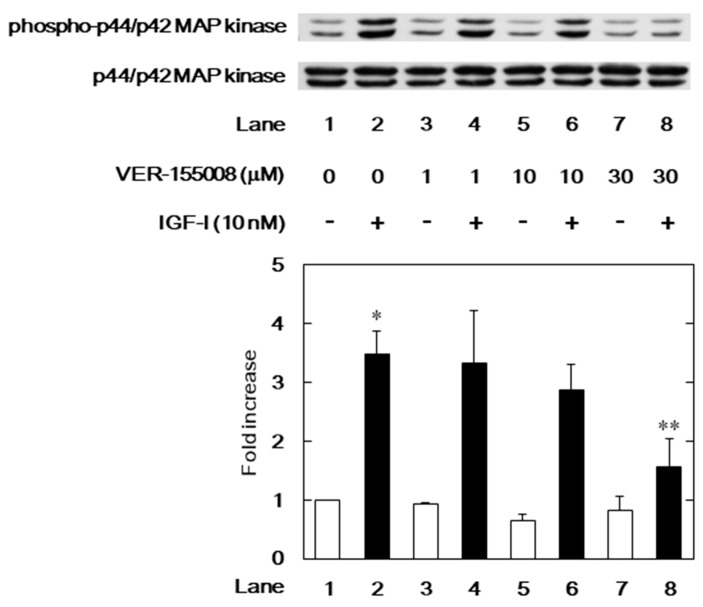
Effect of VER-155008 on the IGF-I-induced p44/p42 MAP kinase phosphorylation in MC3T3-E1 cells. The cells were pretreated with various doses of VER-155008 for 60 min and then stimulated by 10 nM of IGF-I or vehicle for 5 min. Western blot analysis was performed using phospho-specific p44/p42 MAP kinase or p44/p42 MAP kinase antibodies. The histogram shows quantitative representation of the levels of IGF-I-induced phosphorylation obtained from a laser densitometric analysis of three independent cell cultures. The white and black bars indicate IGF (−) and IGF (+), respectively. * *p* < 0.05 compared to the value of the control cells without IGF-I stimulation. ** *p* < 0.05 compared to the value of IGF-I alone.

**Figure 6:**
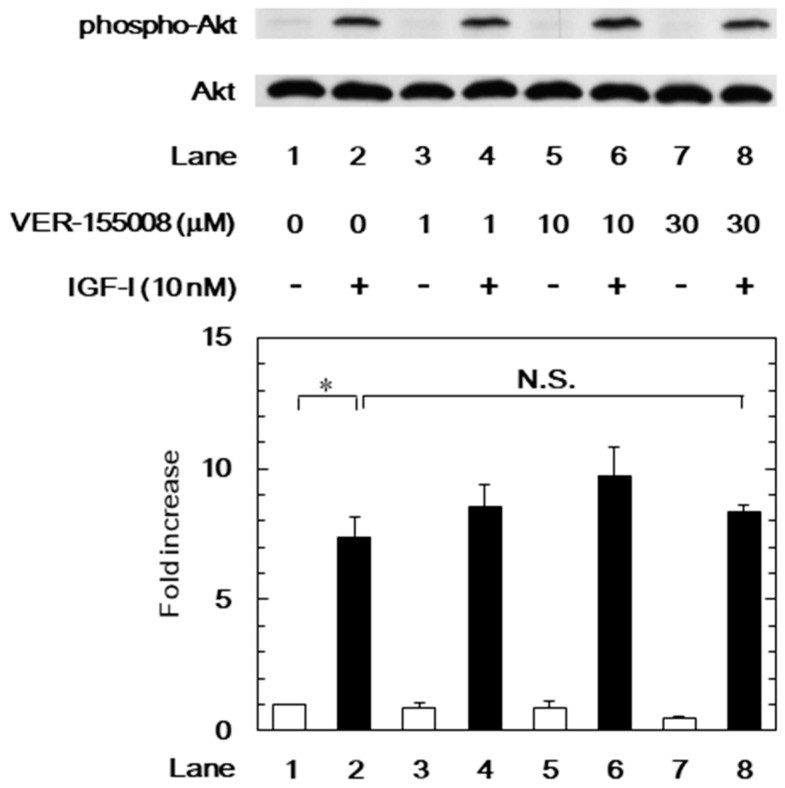
Effect of VER-155008 on the IGF-I-induced phosphorylation of Akt in MC3T3-E1 cells. The cells were pretreated with various doses of VER-155008 for 60 min and then stimulated by 10 nM of IGF-I or vehicle for 3 min. Western blot analysis was performed by using phospho-specific Akt or Akt antibodies. The histogram shows quantitative representation of the levels of IGF-I-induced phosphorylation obtained from a laser densitometric analysis of three independent cell cultures. * *p* < 0.05 compared to the value of the control cells without IGF-I stimulation. The white and black bars indicate IGF (−) and IGF (+), respectively. N.S. designates no significant difference between the indicated pairs.

We have previously demonstrated that IGF-I elicits migration of osteoblast-like MC3T3-E1 cells through the activation of p44/p42 MAP kinase and phosphatidylinositol 3-kinase/Akt [11]. In order to investigate the mechanism underlying the suppression by the HSP70 inhibitor of the IGF-I-stimulated cell migration, we further examined the effects of VER-155008 on the IGF-I-induced phosphorylation of p44/p42 MAP kinase or Akt. The IGF-I-induced phosphorylation of p44/p42 MAP kinase was significantly reduced by VER-155008 (Figure 5). However, VER-155008 failed to affect the IGF-I-induced phosphorylation of Akt (Figure 6).

## 4. Discussion

In the present study, we investigated the effects of HSP70 inhibitors on the IGF-I-elicited migration of osteoblast-like MC3T3-E1 cells. We first examined whether VER-155008, which is an HSP70 inhibitor [22], affects the IGF-I-elicited migration of osteoblast-like MC3T3-E1 cells evaluated by a wound-healing assay. VER-155008 significantly suppressed the IGF-I-elicited migration of MC3T3-E1 cells. In addition, we examined the effect of VER-155008 on the migration induced by IGF-I using a Boyden chamber and demonstrated that the IGF-I-induced migration was reduced by VER-155008. We next examined the effect of YM-08, which is another inhibitor of HSP70 [23], on the IGF-I-elicited migration of MC3T3-E1 cells. YM-08 significantly repressed the migration induced by IGF-I and evaluated by both a wound-healing assay and a transwell cell migration assay. Considering our findings, it is probable that the HSP70 inhibitor suppresses the IGF-I-induced migration of osteoblast-like MC3T3-E1 cells, which suggests that HSP70 acts as a positive regulator in the cell migration. 

With regard to the intracellular signaling of IGF-I in osteoblasts, we have previously shown that p44/p42 MAP kinase and phosphatidylinositol 3-kinase/Akt act as positive regulators in the IGF-I-stimulated migration of osteoblast-like MC3T3-E1 cells [11]. Afterward, we investigated the exact mechanism behind the suppression by the HSP70 inhibitor of the IGF-I-stimulated migration. We demonstrated that the phosphorylation of Akt induced by IGF-I was not affected by VER-155008 in these cells. Thus, it seems unlikely that phosphatidylinositol 3-kinase/Akt is involved in the suppression by the HSP70 inhibitor of IGF-I-induced MC3T3-E1 cell migration. On the contrary, VER-155008 significantly reduced the phosphorylation of p44/p42 MAP kinase induced by IGF-I. Taken together, it is most likely that the HSP70 inhibitor reduces IGF-I-induced migration of osteoblast-like MC3T3-E1 cells through the inhibition of the p44/p42 MAP kinase. Regarding the mechanism of the molecular action of HSP70 on the p44/p42 MAP kinase pathway, it has been reported that mortalin, which is a member of the HSP70 family, could regulate the activity of MEK1/2, which is an upstream kinase of the p44/p42 MAP kinase, via protein phosphatase 1α in human melanoma cells [24]. It is possible that HSP70 could strengthen IGF-I-induced p44/p42 MAP kinase through stabilization of the MEK1/2-phosphorylated status in osteoblasts, which leads to the upregulation of migration. 

Osteoblasts migrate to the sites resorbed by osteoclasts and the migrated osteoblasts then start bone formation at the resorbed sites [3,4,5]. Adequate migration of osteoblasts is indispensable for the regulation of physiological bone remodeling and the appropriate osteoblast migration is considered to be essential for maintaining both the quantity and quality of bone mass. Additionally, the osteoblast migration is crucial in pathological bone metabolic diseases including osteoporosis and fracture repair [3,4,5]. Since HSP70 plays an important role in the survival of cancer cells, HSP70 inhibitors have been developed as anti-cancer agents [25,26]. Our present findings strongly suggest that the HSP70 inhibitor could reduce the IGF-I-elicited migration of osteoblasts. It is established that IGF-I embedded in the bone matrix plays a crucial role in the regulation of bone metabolism [6,7]. Thus, using HSP70 inhibitors as anti-cancer agents, it is possible to modulate bone metabolism to result in a detrimental effect on bone. On the other hand, osteosarcoma is known to be a highly metastatic bone tumor [27]. The metastatic sequence involves migration from the primary tumor site to the surrounding extracellular matrix, intravasation, and extravasation. It has recently reported that overexpression of ribosomal protein L3, which is a target of 5-FU, reduces migration and reciprocally promotes apoptosis of lung and colon cancer cells under the treatment of 5-FU [28,29]. It is likely that suppression of migration causes a benefit for anti-cancer agents such as 5-FU, which has been used for osteosarcoma [30]. It is recognized that HSP70 is potently expressed in human osteosarcoma [31]. It has been reported that VER-155008 reduces cell viability and increases apoptosis of canine osteosarcoma cells [32]. Taking into account our present findings, it is possible that HSP70 inhibitors are useful candidates for drug combination in the chemotherapy of osteosarcoma and may result in the inhibition of tumor metastasis and invasion.

Regarding the expression of HSP70 in osteoblasts, we have previously demonstrated that HSP70 is highly expressed in osteoblast-like MC3T3-E1 cells without stimulation [33]. It has been reported that IGF-I reduces HSP70 expression in macrophages but not in fibroblasts [34]. The effect of IGF-I on the expression of HSP70 in osteoblasts needs to be clarified. On the other hand, we found that HSP70 inhibitors alone did not affect the baseline of osteoblast migration. Thus, it is likely that HSP70 inhibitors hardly affect the osteoblast migration under unstimulated conditions. However, clarification is needed regarding whether HSP70 plays a role in osteoblast migration in general. Further investigations including overexpression of HSP70 in osteoblasts are necessary to clarify the exact roles of HSP70 in bone metabolism.

Taken together, our results strongly suggest that the HSP70 inhibitor reduces the IGF-I-elicited migration of osteoblasts through the suppression of the p44/p42 MAP kinase pathway.
